# Antegrade intramedullary fixation for adolescent fifth metacarpal neck fracture and its impact on epiphyseal growth

**DOI:** 10.1186/s12891-021-04436-w

**Published:** 2021-06-15

**Authors:** Xiao-Lei Fan, Jian Wang, De-Hua Zhang, Feng Mao, Yi Liao, Rui Xiao

**Affiliations:** 1 Department of Orthopaedics, Xiangya Hospital, Central South University, 410008 Changsha, China; 2 Department of Orthopaedics, Karamay Central Hospital of Xinjiang, NO.67 Zhungeer Road, Karamay District, 834000 Karamay, China; 3 Department of Surgical Center, Karamay Central Hospital of Xinjiang, 834000 Karamay, China; 4 Karamay District Center for Disease Control and Prevention , 834000 Karamay, China

**Keywords:** Adolescent, Intramedullary fixation, Epiphyseal closure, Kirschner wire, Metacarpal neck fractures

## Abstract

**Background:**

Antegrade intramedullary nailing (AIMN) with Kirschner wire (K-wire) is a minimally invasive osteosynthesis technique. This procedure has been widely performed to treat the fifth metacarpal neck fracture (FMNF) in adults. This study was performed to determine whether using AIMN with a single K-wire to treat FMNF in adolescents would have good clinical and radiographic outcomes.

**Methods:**

In this retrospective study, 21 children (aged 11–16 years) with FMNF were treated using AIMN with a single K-wire from May 2017 to January 2020 in our hospital. Indications for intervention were severe displacement with malrotation deformity, apex dorsal angulation of greater than 40°, or both. Collected data included apex dorsal angulation, range of motion (ROM) in the fifth metacarpophalangeal (MCP) joint, Visual Analog Scale (VAS) for pain, grip strength, and Disabilities of the Arm, Shoulder, and Hand (DASH) score.

**Results:**

All patients were followed up for 12–24 months (average, 16.57 months), and all patients obtained anatomical reduction postoperatively. The healing time was 2.69 ± 0.83 months (range, 2–4 months). Average apex dorsal angulation was reduced significantly from 44.49°±2.64° to 15.74°±2.47° (*P* < 0.001). The average ROM in the MCP joint and apex dorsal angulation of the injured side were not significantly different from those of the uninjured side. The average DASH score was 1.76 ± 1.48 (range, 0–4), the mean VAS was 0.19 ± 0.60 (range, 0–2), and the mean grip strength was 91.55 %±4.52 % (range, 85–101 %). No secondary displacement, dysfunction, nonunion, infection, or osteonecrosis was observed during the follow-up. Although premature epiphyseal closure was found in one patient, no long-term clinical finding of angulation or shortening was identified.

**Conclusions:**

Antegrade intramedullary fixation with single K-wire was an effective and reliable technique that successfully resulted in good functional and cosmetic outcomes for treating adolescents with FMNF. The impact on the growth plate was low in this population given that most patients were at or approaching skeletal maturity.

**Level of evidence:**

Level IV.

**Supplementary Information:**

The online version contains supplementary material available at 10.1186/s12891-021-04436-w.

## Background

Metacarpal fractures account for 10–39 % of all hand fractures in children, especially in the age group of 13–16 years [1, 2]. The most common type of metacarpal fracture is the so-called boxer’s fracture, which involves the neck of the ring or small finger metacarpal. These fractures are frequently observed in active adolescent males and occur in the dominant hand. Fifth metacarpal neck fracture (FMNF) is a typical injury caused by aggressive behavior, with sport-related injuries or hitting a hard object found to be the predominant cause [3, 4]. The majority of these fractures can be successfully treated nonoperatively because of a potent remodeling ability and rapid healing of the growing bone by using immobilization with or without reduction [5–7]. When deformities, such as apex dorsal angulation of greater than 40°, shortening by more than 5 mm, or malrotation, are present, surgical correction is necessary to prevent significant malunion that can lead to poor cosmetic or functional outcomes [8–10]. Malrotation or apex dorsal angulation of the metacarpal frequently causes a reduction in the grip strength and a lack of full extension of the fifth finger [11, 12].

Many surgical techniques have been described to treat the FMNF to achieve reduction and obtain stability. Some of these techniques are antegrade intramedullary nailing (AIMN), retrograde intramedullary pinning by using Kirschner wire (K-wire), transverse pinning with K-wire, external fixation, and standard or low-profile plates [7, 11]. The surgical technique should be selected by considering how to minimize soft-tissue disruption and allow early motion. AIMN with a K-wire is a minimally invasive osteosynthesis technique that offers a simple operation and reliable fixation of the FMNF, producing better hand function outcomes than other processes [13]. The AIMN with K-wire has been frequently performed in the past decade and has good clinical outcomes in adults [14]. We determined whether using AIMN with K-wire to treat adolescents with displaced FMNF would present similar results.

## Methods

### Patients

This study was performed under the ethical standards of the 1964 Declaration of Helsinki. Fifty-two consecutive adolescents with FMNF were treated from May 2017 to January 2020 in our hospital. Twenty-one of these patients with FMNF required surgical intervention and were included in the study. Inclusion criteria were the presence of rotational displacement deformity, apex dorsal angulation of greater than 40°, or both. The patients were treated with closed reduction and single antegrade intramedullary K-wire fixation. The other 31 patients, who were managed nonoperatively or treated with closed reduction and plaster cast external fixation, were excluded from this study.

The patients included seventeen males and four females. The general characteristics of the patients before surgery are shown in Table 1. One patient suffered fourth and fifth metacarpal neck fractures. A total of 22 metacarpal neck fractures were treated. Seventeen patients were injured because of their direct punching of a hard surface, while four subjects had a falling incident. The apex dorsal angulation of the metacarpal neck (Table 1) was measured on the oblique X-rays in accordance with a previous study by Picture Archiving and Communication Systems [15]. The Medullary Canal method in the pronated 45° oblique view was used.

**Table 1:** Patient Demographics

	Mean (Range)
Age, year	13.30 ± 1.35(11–16)
Sex, male/female	17/4
Dominant hand, yes/no	15/6
Injured side, left/right	3/18
Apex dorsal angulation, °	44.49 ± 2.64˚(40.5–49.8)

Measurement data are presented as mean ± standard deviation

### Surgical procedure

Procedures were performed under brachial plexus block or general anesthesia and by experienced surgeons using fluoroscopy. According to the preoperative width of the medullary canal, a K-wire with the appropriate diameter (1.5–1.8 mm) was chosen. Approximately 0.5-1 cm of the tip of the K-wire was bent slightly by 10–20° for use as a joystick to control the fragments. The opposite end of the wire was roughly bent in the same plane and fashioned as a joystick handle. A simple transverse incision of approximately 0.5 cm was made at the distal of the carpometacarpal joint, proximal to the fifth metacarpal. Blunt dissection down to the bone was performed to avoid damage to the dorsal branch of the ulnar nerve. A cortical window of entry point was created with the sharp end of the same size K-wire under the C-arm radiographic guidance, and then a pre-bent K-wire was inserted. Gentle oscillating movements were used to insert the K-wire into and advanced along the medullary canal until near the fracture site. The surgeon reduced the fracture by using the Jahss maneuver [16]. Once reduction was confirmed, the K-wire was advanced across the fracture site to the subchondral bone. The K-wire was first advanced toward the palmar region and then rotated toward the dorsum upon reaching the metacarpal head to further correct the dorsal angulation of the metacarpal neck. The base of the K-wire was bent and cut below the subcutaneous tissues, and incisions were closed by using absorbable suture.

### Post-operative management and follow-up

A short-arm plaster cast was applied in all cases for 2 weeks to prevent any secondary displacement of the fracture applied in the “safe” position [17]. Patients were instructed to begin active interphalangeal exercises with a splint at 1 day or 2 days after surgery. The Xray examination of the hand was performed for review in the first week, every month for the first 3 months, every 3 months for the first year, and two years postoperatively. Once the plaster cast was removed after 2–3 weeks, the functional exercise of the metacarpophalangeal (MCP) joints and interphalangeal joints was performed gradually. After the radiographic confirmation of fracture union, the K-wires of all patients were removed under local anesthesia or general anesthesia at 4–6 weeks postoperatively. The procedure was from the original incision, sometimes under fluoroscopy. Full weight-bearing was not allowed, until the fractures showed bony healing.

Any postoperative complications, such as dysfunction, residual pain, secondary displacement, infection, and osteonecrosis, were recorded during the follow-up. At 12 months after surgery, we evaluated the range of motion (ROM) of the MCP, grip strength, Visual Analog Scale (VAS) for pain, and Disabilities of the Arm, Shoulder, and Hand (DASH) score (0–100 points) [18] from the clinical point of view. Grip strength values are expressed as percentages of those of the contralateral hands. Anteroposterior and oblique X-rays were taken to assess for residual deformity.

### Statistical analysis

Statistical analysis was performed using the SPSS software package (SPSS, Chicago, IL, USA). The measurement data were expressed as mean ± standard deviation and analyzed using a paired t-test. The χ^2^-test was used to check the counted data. Statistical significance was set to *P* < 0.05.

## Results

**Fig. 1:**
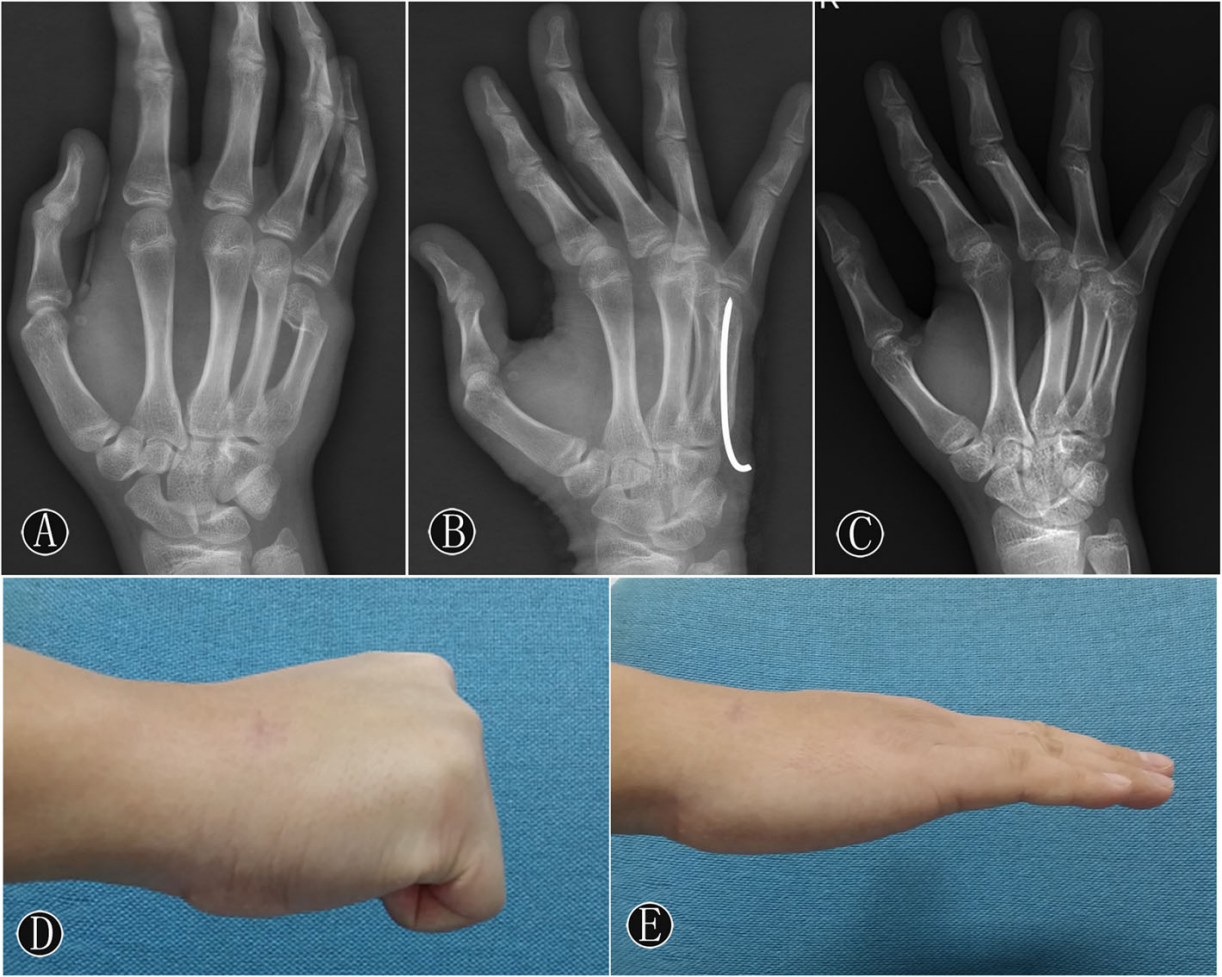
Male, 13 years old, FMNK of right the hand caused by falling. **A** The anteroposterior image showed the apex dorsal angulation preoperatively. **B** The oblique image showed a three-point supporting role of k-wire in the head, body, and tail, and the apex dorsal angulation was completely corrected one month after surgery. **C** After removal of the k-wire postoperatively, oblique images showed good fracture healing. **D** and **E** The general images of lateral position showed excellent flexion and extension function of MCP joints.

**Fig. 2:**
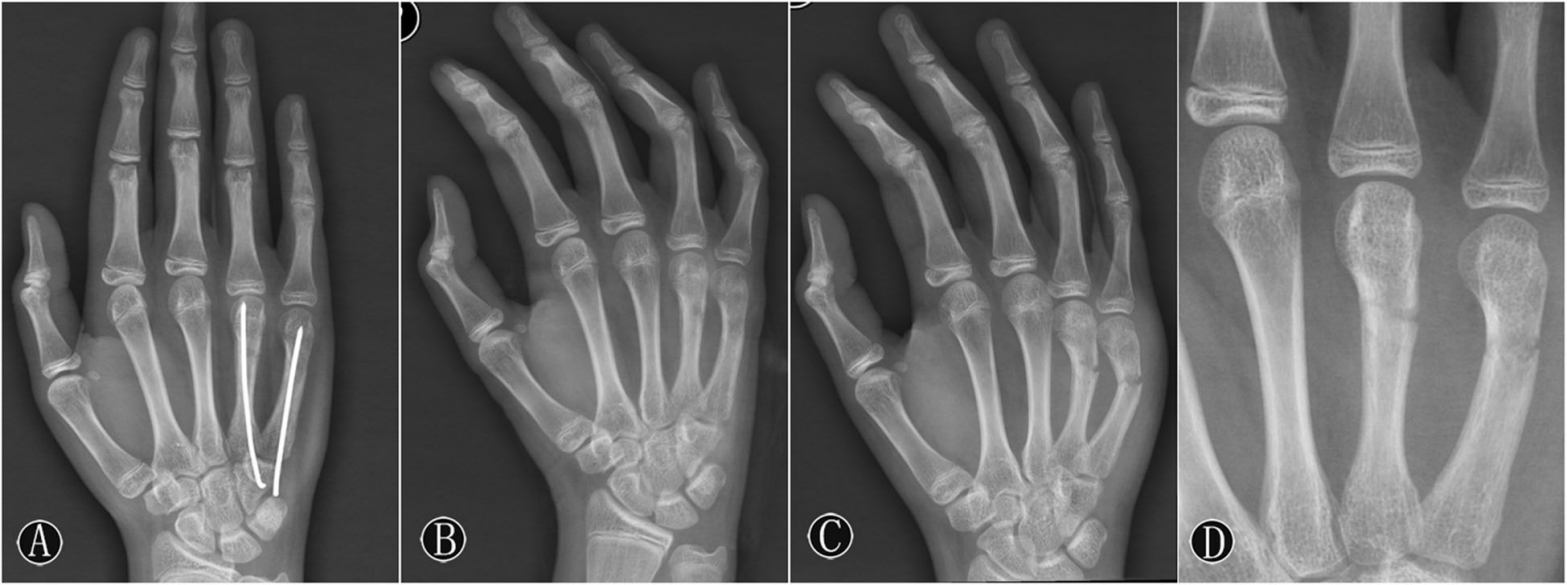
The patient with premature epiphyseal closure: male, 12 years old, fourth and fifth metacarpal neck fracture of the right hand. **A** No epiphyseal closure was found in the anteroposterior image postoperatively. **B** After removal of the k-wire, X-rays showed that the angulation of the metacarpal was completely corrected. **C** Oblique images showed a second fracture of the fourth, fifth metacarpal after 4 months. **D** Local magnification radiograph indicated premature epiphyseal closure.

All patients were followed up for 12–24 months (average, 16.57 months). A typical case is shown in Fig. 1. At 3 months postoperatively, no patient had evidence of injury to the sensory branch of the ulnar nerve, infection, or skin healing problems. Fractures in all patients were healed (Table 2), with no cases of secondary displacement or rotational malunion. However, premature epiphyseal closure was found in the patient who had fourth and fifth metacarpal neck fractures (Fig. 2), when he had a second fracture at 4 months after surgery. Given the potential risk of physeal arrest, the second fracture was treated with closed reduction and immobilization by using a splint. The diameters of the K-wire and epiphyseal plate were measured in all patients. The relative size of the cross-sectional area was expressed by the size of the K-wire versus the overall size of the physis as percentages (Table 2). The relative epiphyseal entry site and trajectory of the K-wire were observed. No significant difference was observed between the premature epiphyseal closure and the relative entry site of the K-wire (Table 3; *P* = 0.567).

**Table 2:** Post-operative Outcomes

	Mean (Range)
Fracture healing time, months	2.69 ± 0.83 (2–4)
Cross-sectional area, %	3.46 ± 0.78 (2.1–4.9)
Premature epiphyseal closure, yes/no	1/20
DASH	1.76 ± 1.48 (0–4)
VAS	0.19 ± 0.60 (0–2)
Grip strength of opposite side, %	91.55 ± 4.52 (85–101)

Measurement data are presented as mean ± standard deviation

**Table 3:** Relative Entry Site of Physis and Premature Epiphyseal Closure

	Premature epiphyseal closure		
K-Wire	No	Yes	Total
Centrally	13	1(fourth)^b^	14
Peripherally	7	1(fifth)^b^	8
Total	20	2	22^a^

^a^Twenty-two cases of metacarpal fractures include the fourth metacarpal fractures of the double fracture patient. Fisher’s exact test was used for counting data

^b^The same person

At 12 months postoperatively, only one patient reported residual pain of the MCP joint during daily activity. VAS, DASH score, and grip strength were collected (Table 2). The apex dorsal angulation of the affected side was 44.49°±2.64° preoperatively and 15.74°±2.47° postoperatively, indicating a significant improvement (*P* < 0.001). The average ROM of the fifth MCP joint of the affected side was 87.97°±2.35°, and no significant difference was observed compared with that of the unaffected side (88.45°±2.39°, *P* = 0.084). No significant difference in the apex dorsal angulation was found between the affected and unaffected sides (15.74°±2.47° vs. 15.17°±1.70°; *P* = 0.056) (Table 4). At the final follow-up, all patients achieved excellent functional results.

**Table 4:** Apex Dorsal Angulation of Fifth Metacarpal, and ROM of MCP Joints

	ROM of MCP joint,°	Apex dorsal angulation (Head‑shaft angle)		*P*-value
		Pre-operative,°	Post-operative, °	
Affected	87.97±2.35˚	44.49±2.64˚	15.74±2.47˚	<0.001
unaffected	88.45±2.39˚	-	15.17±1.70˚	-
*P*-value	0.084	-	0.056	-

## Discussion

Most current studies have suggested that AIMN is a simple and effective method of treating metacarpal fractures and has a relatively low possibility of complications [19–21]. This procedure has been emerging as the commonly accepted technique for treating FMNFs. However, the acceptable range of apex dorsal angulation deformity in the fifth metacarpal is controversial. Most scholars believe that more than 40° at the small finger is likely to be poorly tolerated and is an indication for surgery [11, 22]. Some studies have reported an acceptance of up to 70° of angulation at the small metacarpal neck [23]. Notably, angulation greater than 30° has been associated with a decrease in the flexor digiti minimi grip strength and ROM [11, 12]. Therefore, in this study, the apex dorsal angulation greater than 40° was applied for surgical indication. Besides, several studies have described the reliability of the oblique view in the measurement of angulation [24].

This retrospective study was aimed to assess the clinical and radiographic results of using AIMN with single K-wire in adolescents with FMNF. Through follow-up, we found that all 21 adolescents showed minimized soft-tissue disruption and largest total active motion of hand. The absence of a surgical wound around the MCP joint gave patients a greater initiative to perform early function exercises to avoid adhesion and stiffness. The active ROMs of the fifth MCP on both hands were almost the same. In comparison to the contralateral hand, almost no apex dorsal angular malformation was found. All outcomes were in close agreement with some previous studies, in which elastic intramedullary nailing was used to treat adolescents with FMNF [9, 25]. This technique is well-suited to the small diameter of the medullary canal in adolescents. Elastic intramedullary nails are more expensive and have a limited range of diameters. By contrast, the K-wire is widely used in clinical practice and inexpensive and has a wide option for diameters.

The K-wire placed across the physis has been reported to probably contribute to physeal arrest when the physis was violated by hardware [26]. Factors, such as size, location, number of passes, and number of K-wires, may contribute to physeal injury at the time of surgery. Physeal bar formation mainly contributes to complications in the epiphyseal growth. A previous animal study has suggested that drill injury of 7–9 % of the cross-sectional area of the physis was enough to result in a growth disturbance [27]. Small central disruptions of the physis are not perceived to alter the physeal growth [28]. However, William et al. found that the K-wire across the physis centrally and peripherally appeared to have the same risk for physeal bar formation [29]. Dorman et al. found that K-wire fixation did not increase the risk of growth arrest, and physeal injuries at the end of growth did not change the limb axis [30]. No consensus has been reached regarding the impact of a K-wire on physeal injury.

Premature epiphyseal closure caused by physeal arrest has a high incidence in the distal femoral and distal radius physeal fractures [26, 31, 32]. However, the impact of a K-wire on the metacarpophalangeal physeal is rarely reported. In the patient who had premature epiphyseal closure, the drill injuries of the cross-sectional area of the physis were only 3.7 and 3.9 % in the fourth and fifth metacarpals, respectively. The relative entry site was peripherally and centrally in the fourth and fifth metacarpal physis, respectively (Table 3). After the removal of the K-wire, no obvious physeal bar formation was observed. Even so, we could imagine that premature epiphyseal closure might be associated with multiple attempts and passes of the K-wire across the physis. We cannot ascertain whether the physeal injury affected by K-wire, resulted in premature epiphyseal closure. Further research is needed to determine the impact of a K-wire on epiphyseal growth. More cases and studies are required to provide clinical guidance for treating FMNF in adolescents.

Although premature epiphyseal closure was found in one patient (4.8 %), no long-term clinical findings of angulation or shortening was identified. Moreover, during follow-up, almost all adolescent patients had normal epiphyseal closure within 1–2 years after surgery. The risk of the closure of the growth plate may be small, given that most adolescents are at or approaching skeletal maturity. Children younger than 11 years or with strong epiphyseal growth ability can experience premature epiphyseal closure when treated with AIMN. Closed reduction and immobilization with a splint or cast is the first choice[6, 33]. If deformities, such as apex dorsal angulation, shortening, and malrotation, are difficult to reduce, transverse pinning may be used to avoid passing through the physis [34]. For adolescents at or approaching skeletal maturity, AIMN with K-wire can be safely used to treat FMNF.

Our study has limitations, because it is a retrospective study that included a relatively small number of patients, without a control series of patients treated with a different method. However, the results proved that AIMN with single K-wire is a straightforward, reliable, and minimally invasive surgical technique for treating FMNF in adolescents.

## Conclusions

The adolescent population is the age group with a high incidence of metacarpal neck fracture. AIMN with single K-wire is an effective and readily available method for treating FMNF in adolescents. The results confirmed minimal impact on the growth plate and satisfactory functional outcomes.

## Supplementary Information

**Additional file 1.**

## Data Availability

The datasets used and/or analysed during the current study are available from the corresponding author on reasonable request.

## Abbreviations

AIMN: Antegrade intramedullary nailing
K-wire: Kirschner wire
FMNF: Fifth metacarpal neck fracture
ROM: Range of motion
MCP: Metacarpophalangeal
VAS: Visual Analog Scale
DASH: Disabilities of the Arm, Shoulder, and Hand
